# Electromagnetic Interference Shielding of 2D Transition Metal Carbide (MXene)/Metal Ion Composites

**DOI:** 10.3390/nano11112929

**Published:** 2021-11-01

**Authors:** Xuefeng Xia, Quanlan Xiao

**Affiliations:** International Collaborative Laboratory of 2D Materials for Optoelectronics Science and Technology of Ministry of Education, Institute of Microscale Optoelectronics, Shenzhen University, Shenzhen 518060, China; xiaxuefeng2018@email.szu.edu.cn

**Keywords:** MXene Ti_3_C_2_, electromagnetic interference, Fe^3+^/Co^2+^/Ni^2^^+^

## Abstract

In this work, Ti_3_C_2_, which has a loosely packed accordion-like structure in transition metal carbide (MXene) form, is fabricated and adsorbed by three metal ions (Fe^3+^/Co^2+^/Ni^2+^). The electromagnetic interference (EMI) shielding performance of Ti_3_C_2_ and Ti_3_C_2_:Fe^3+^/Co^2+^/Ni^2+^ films is researched in detail, demonstrating that the EMI shielding effectiveness can be improved by adsorbing by Fe^3+^/Co^2+^/Ni^2+^ ions because the metal ion adsorbing can improve the absorption efficiency via electromagnetic wave scattering. The studied Ti_3_C_2_:Fe^3+^/Co^2+^/Ni^2+^ films can be used as good EMI shielding materials for communications, electronics, military, and other applications.

## 1. Introduction

In recent years, due to the continuous development of science and technology, newer and faster wireless communication networks have been further covering the entire world, and the shrinking of the volume of intelligent electronic devices throughout the world has attracted the attention of people in the field of electromagnetic interference shielding. Because electromagnetic radiation not only causes harm to the human body but also affects the normal use of electronic equipment, and the traditional electromagnetic interference shielding material has difficulty coping with the increasingly sophisticated and miniaturized electronic equipment, the search for new electromagnetic interference shielding materials has become a focus [1,2,3,4,5,6,7,8,9]. Many articles have studied the electromagnetic shielding effectiveness of some composite materials [10,11,12,13,14,15,16,17,18,19]. For example, Bhawal P. et al. [11]. used different mass fractions of CNFs to improve the electromagnetic shielding effectiveness. Ling J. et al. [13]. studied microcellular polyetherimide/graphene composite foams as heat-resistant electromagnetic shielding materials. Li N. et al. [15]. studied single-walled carbon nanotube (SWNT)-polymer composites as excellent lightweight electromagnetic shielding materials. Compared with these polymer materials, research on the electromagnetic shielding effectiveness of two-dimensional materials is limited. Therefore, we use three different ion solutions with the same concentration to study the electromagnetic shielding effectiveness of MXene films doped with different ions. An MXene is a two-dimensional material obtained by chemically etching the A layer of an MAX phase material [20,21,22,23,24,25]. The M in the MAX phase generally represents an early transition metal, A represents a group XIII or XIV element, and X represents C or N [26,27,28,29,30,31,32,33]. An MXene has low density and high flexibility compared with traditional electromagnetic interference shielding materials and can be well applied in some delicate and tiny environments.

Because an MXene can exhibit a loose-layered structure that causes multiple internal reflections of electromagnetic waves, electromagnetic waves can be dissipated as thermal energy in the material, which contributes greatly to electromagnetic interference shielding [34,35,36,37,38,39,40]. In addition, metal is the originally used filler of electromagnetic interference shielding materials. It has excellent electrical conductivity. Metal ions inherit this property, and their dispersion in polymers is good and does not affect the original mechanical properties of the material [41]. For example, Li^+^ ions and K^+^ have been used as intercalants to improve the conductivity of two-dimensional materials [42]. Regarding the surface modification of Ti_3_C_2_, some metal ions enhance the internal scattering, further enhancing the shielding effectiveness for electromagnetic interference. This method also does not increase the volume or quantity of the shielding material, and the shielding effect can be well improved by embedding a small amount [43,44,45,46].

Herein, we used this method to obtain Ti_3_C_2_ with an accordion-like structure by etching Ti_3_AlC_2_ with hydrofluoric acid (HF). Ti_3_C_2_/metal ion composite materials were obtained by reacting Ti_3_C_2_ with Fe^3+^/Co^2+^/Ni^2+^ ion solutions, and Ti_3_C_2_ and Ti_3_C_2_:Fe^3+^/Co^2+^/Ni^2+^ films were obtained by vacuum filtration. Finally, the electromagnetic interference shielding performance of the four films at frequencies of 8–18 GHz was tested by the coaxial method. The results show that the electromagnetic interference (EMI) shielding effectiveness can be improved to different degrees when Ti_3_C_2_ is adsorbed by Fe^3+^/Co^2+^/Ni^2+^ ions, and the most obvious effect is observed for Ti_3_C_2_ adsorbing by Fe^3+^ ions. This is due to the fact that metal ions adsorption can enhance the conductivity of the materials and improve the absorption effectiveness via electromagnetic wave scattering in the materials. Such EMI shielding materials have a variety of potential applications, such as in communications, electronics, and the military.

## 2. Methods

### 2.1. Material Synthesis

The starting materials were Ti_3_AlC_2_ (99.5%, Mingshan New Materials, Guangzhou, China), hydrofluoric acid (HF, 40 wt%, Macklin, Shanghai, China), ethanol (C_2_H_5_OH, 99.5%, Hushi, Shenzhen, China), isopropanol (C_3_H_8_O, 99.5%, Macklin, Shanghai, China), Fe(NO_3_)_3_/Co(NO_3_)_2_/Ni(NO_3_)_2_ (Homemade), and deionized water (DI, Homemade).

#### 2.1.1. Preparation of Ti_3_C_2_ Powder

In this paper, Ti_3_C_2_ was obtained by selective etching of Ti_3_AlC_2_ using HF. First, 0.2 g of Ti_3_AlC_2_ powder was slowly added to 40–50 mL of HF solution in a centrifuge tube in a fume hood (to prevent violent reaction), which were well mixed at a constant temperature of 40 °C and 300 rpm (to prevent liquid splash), and they were fully reacted for approximately 20 h. Then, the mixture was rinsed with deionized water several times until the pH became neutral and dried at 60 °C for 5–8 h in a vacuum oven to obtain pure Ti_3_C_2_ powder.

#### 2.1.2. Incorporation of Metal Ions

**Figure 1:**
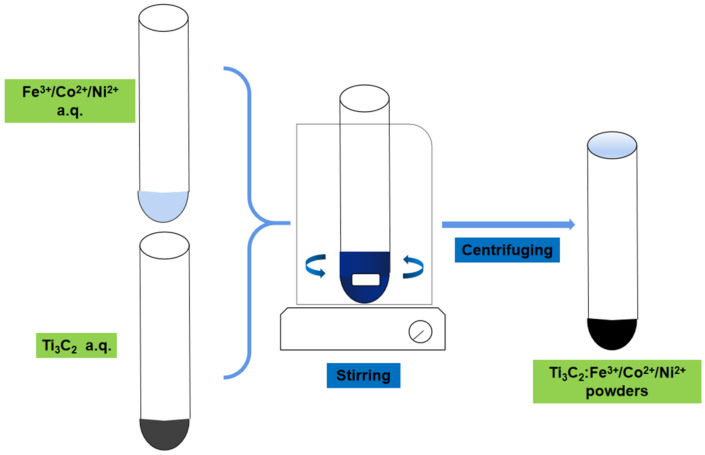
The schematic diagram for the preparation of Ti_3_C_2_:Fe^3+^/Co^2+^/Ni^2+^ powders.

A total of 0.1 g of Ti_3_C_2_ powder was mixed with 10^−5^ mol·L^−1^ Fe(NO_3_)_3_/Co(NO_3_)_2_/Ni(NO_3_)_2_ solutions, and the mixture was sonicated in a water bath for 6 min and stirred at 800 rpm for 3 h. Then, it was centrifuged at 13,000 rpm for 10 min, and the precipitate was collected, washed with DI water several times, and dried at 60 °C for 3 h in a vacuum oven to obtain Ti_3_C_2_:Fe^3+^/Co^2+^/Ni^2+^ powders; the schematic diagram of preparation is shown in Figure 1.

#### 2.1.3. Preparation of Ti_3_C_2_ and Ti_3_C_2_:Fe^3+^/Co^2+^/Ni^2+^ Films

We used a 20 mL Ti_3_C_2_ aqueous solution with a concentration of 5 mg/mL and a filter membrane made of Nylon-66 to fabricate a Ti_3_C_2_ film with a uniform surface by vacuum suction filtration. Similarly, the Ti_3_C_2_:Fe^3+^/Co^2+^/Ni^2+^ films could be obtained using the same method.

### 2.2. Characterization Methods

We used X-ray diffraction (XRD, Bruker-D8 Advance, Jena, Germany) with Cu/Ka (λ = 0.1541 nm) radiation to investigate the crystal structure and phase purity of the powders. We used a scanning electron microscope (SEM, ZEISS-SUPRA55, Berlin, Germany) and a transmission electron microscope (TEM, JEOL-JEM-3200FS, Tokyo, Japan) to observe the morphology, structure, and size distribution of the powders. We studied the composition of the powders by using the SEM equipped with an energy-dispersive X-ray spectrometer (EDS). A high-resolution transmission electron microscope (HR-TEM) and selected area electron diffraction (SAED) patterns were used to further determine the structure and phase of the powders using the JEOL-JEM-3200FS TEM. The Raman spectra were measured with a Raman spectrometer (Renishaw inVia, Gloucestershire, UK), using a 532 nm laser as the excitation source. The thicknesses of Ti_3_C_2_, Ti_3_C_2_/Fe^3+^/Co^2+^/Ni^2+^ films were measured using a micrometer (103–137, Mitutoyo, Tokyo, Japan). The electrical conductivity was measured with a conductivity meter (S230-K, Mettler Toledo, Berne, Switzerland). For testing of the electromagnetic interference shielding effectiveness (EMI SE), we used the near-field test method. The instruments used were a spectrum analyzer (FSV40, R&S), a signal source (SMB100A, R&S), and a near-field probe (langer XF1set, Langer emv-technik, Berlin, Germany). First, the signal source frequency and strength were set for the empty window test. Then, the prepared Ti_3_C_2_ film sample was attached to the sample holder, and the test was started after fixing it. In the same way, Ti_3_C_2_ films doped with the three different metal ions were tested in sequence. All measurements were performed at room temperature.

## 3. Results and Discussion

**Figure 2:**
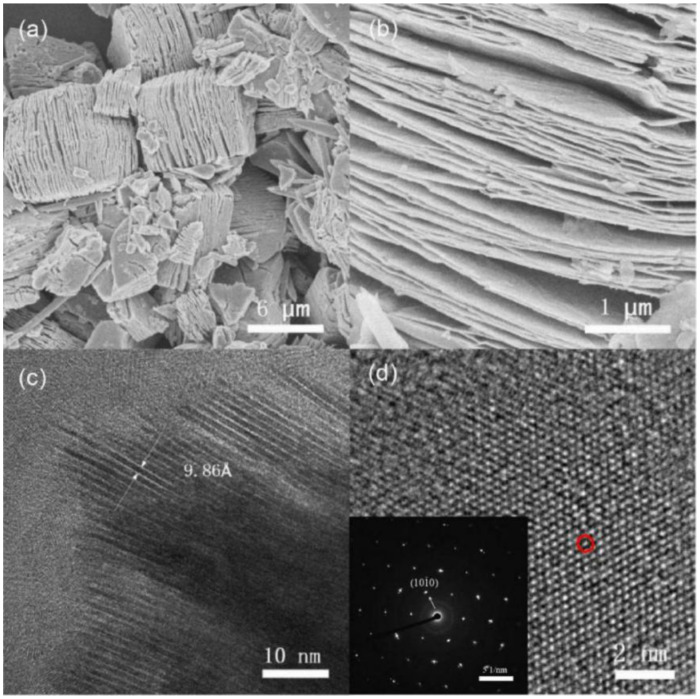
(**a**,**b**) SEM images of layered Ti_3_C_2_ after HF treatment. (**c**) The interlayer distance of layered Ti_3_C_2_ sheets was determined via TEM. (**d**) HR-TEM image of Ti_3_C_2_ and inset showing the corresponding SAED pattern.

Figure 2a,b shows SEM images of layered Ti_3_C_2_ after HF treatment. A large number of accordion-like structures of layered Ti_3_C_2_ can be successfully synthesized by etching with HF, and gaps can be observed among the layers of layered Ti_3_C_2_ in Figure 2b. It can be inferred that the Al atoms in the original MAX phase have been substantially removed. Figure 2c shows a TEM image of layered Ti_3_C_2_. The interlayer distance is determined to be 9.86 Å, which is consistent with the previous theoretical value of 9.93 Å [47]. Figure 2d shows an HR-TEM image of layered Ti_3_C_2_. Parallel and ordered lattice fringes can be observed in the HR-TEM image, revealing the hexagonal symmetric lattice of layered Ti_3_C_2_, which matches the results of previous papers [48]. The *d*-spacing of layered Ti_3_C_2_ is 0.25 nm. Furthermore, the SAED pattern of layered Ti_3_C_2_ shown in the inset in Figure 2d further reveals the single crystal characteristic of layered Ti_3_C_2_.

**Figure 3:**
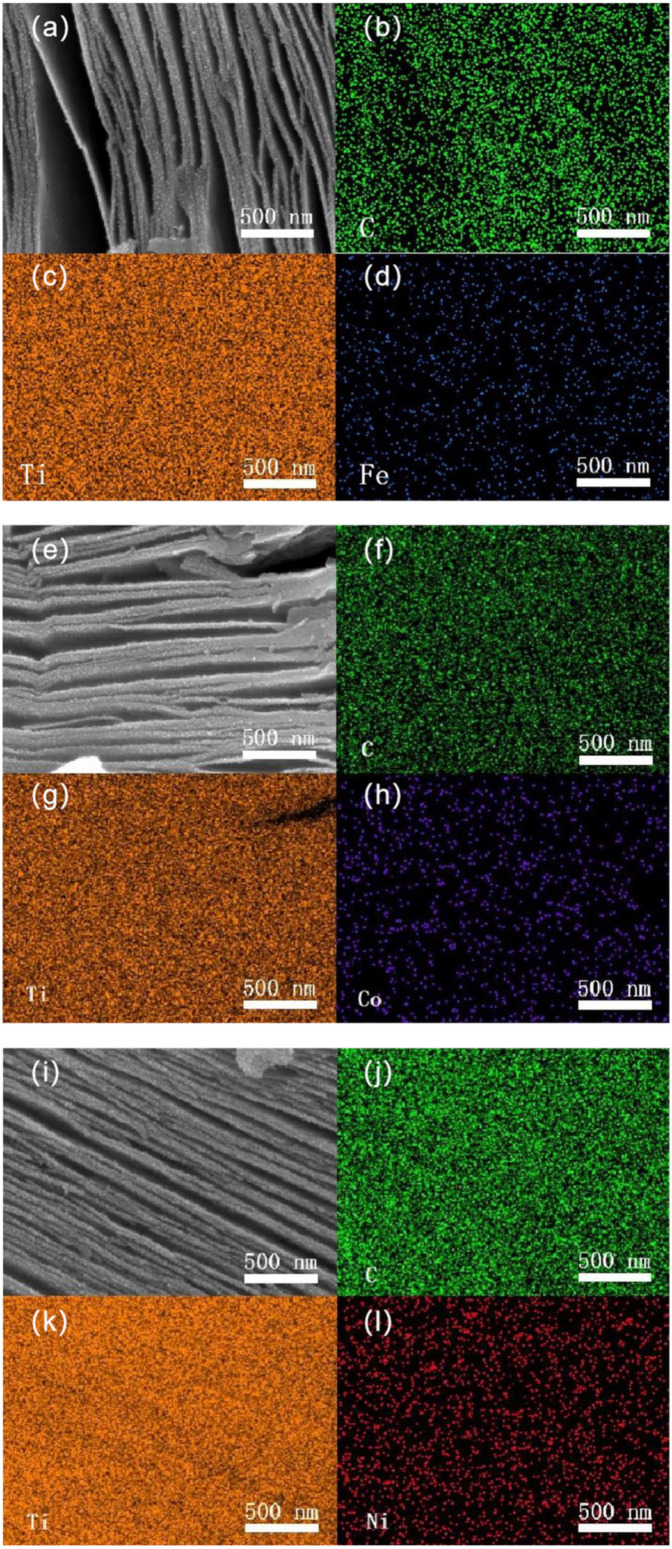
SEM and corresponding EDS mapping images of Ti_3_C_2_/Fe^3+^/Co^2+^/Ni^2+^:Ti_3_C_2_/Fe^3+^ (**a**–**d**), Ti_3_C_2_/Co^2+^ (**e**–**h**), and Ti_3_C_2_/Ni^2+^ (**i**–**l**).

Figure 3 shows SEM and EDS mapping images of Ti_3_C_2_:Fe^3+^/Co^2+^/Ni^2+^. In Figure 3a, we can observe that a large number of nanoparticles appear on the interlayer surfaces of Ti_3_C_2_:Fe^3+^. The EDS mapping images of Fe, Ti, and C are depicted in Figure 3b–d, respectively, which are used to investigate the introduction and distribution of Fe^3+^ ions. Similarly, in Figure 3e–l, we can also observe similar phenomena, and a large number of Co^2+^ and Ni^2+^ ions are introduced into layered Ti_3_C_2_. Therefore, after the reaction of Ti_3_C_2_ and Fe(NO_3_)_3_/Co(NO_3_)_2_/Ni(NO_3_)_2_ solutions, an obvious adsorbing effect occurs, that is, most of the Fe^3+^/Co^2+^/Ni^2+^ ions are distributed on the surface of Ti_3_C_2_, and the others are intercalated into the layers.

**Figure 4:**
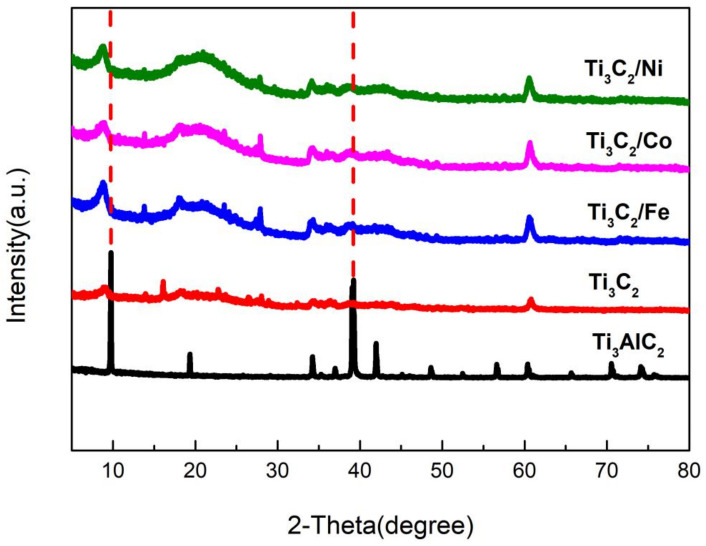
XRD patterns of Ti_3_AlC_2_, Ti_3_C_2_, and Ti_3_C_2_/Fe^3+^/Co^2+^/Ni^2+^ powders.

Figure 4 shows the XRD patterns of Ti_3_AlC_2_, Ti_3_C_2_, and Ti_3_C_2_/Fe^3+^/Co^2+^/Ni^2+^ powders. Compared with the XRD pattern of Ti_3_AlC_2_, the prominent peak at ~39° (the (104) plane) disappears completely after etching with HF, which is due to the removal of Al [20,21,22,23,24,25]. In addition, the peak at ~9° can be attributed to the diffraction of the (002) plane, which has a significant blue shift. According to the XRD data, after Ti_3_AlC_2_ is etched, the diffraction peak attributed to the (002) plane undergoes a large blue shift (from 9.77° to 9.06°). Ti_3_C_2_ adsorbed by Fe^3+^/Co^2+^/Ni^2+^ has different blue shifts (from 9.06° to 8.82°/8.67°/8.76°), and the intensity of the peak increases upon adsorbing by different metal ions. The main reason for this result should be interpreted as the distribution of the different adsorbing ions in the interlayer space of Ti_3_C_2_.

**Figure 5:**
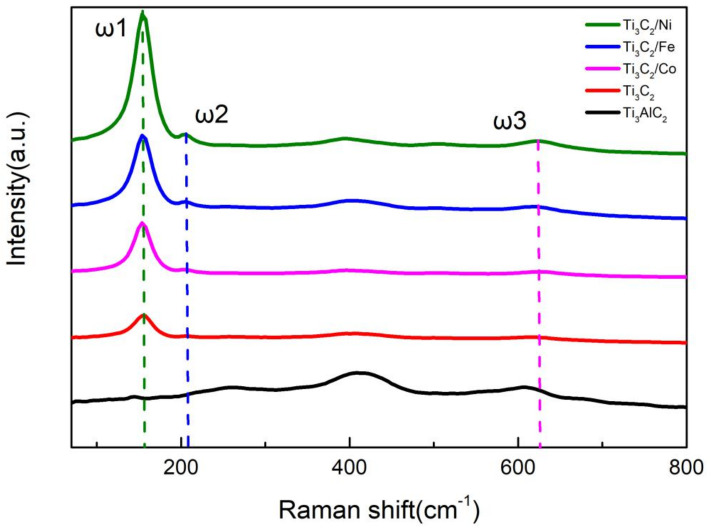
Raman spectra of Ti_3_AlC_2_, Ti_3_C_2_, and Ti_3_C_2_/Fe^3+^/Co^2+^Ni^2+^ powders.

Figure 5 shows the Raman spectra of Ti_3_AlC_2_, Ti_3_C_2_, and Ti_3_C_2_/Fe^3+^/Co^2+^/Ni^2+^ powders. In the Raman spectrum of Ti_3_AlC_2_, two peaks are observed at 280 cm^−1^ and 420 cm^−1^, both of which are derived from the vibration mode of the Al atom. In the Raman spectrum of Ti_3_C_2_, these two peaks have disappeared, demonstrating that the Al atom was completely removed after HF etching. In addition, the peak (ω1) at 155 cm^−1^ is enhanced to different degrees when Fe^3+^/Co^2+^/Ni^2+^ ions are adsorbed into Ti_3_C_2_, and the peak can be observed more clearly. It is indicated that the in-plane motion of Ti2 and C atoms may be enhanced by adsorbing metal ions. The peaks (ω2 and ω3) corresponding to out-of-plane stretching vibrations of Ti2 and C atoms appear at 228 cm^−1^ and 599 cm^−1^, respectively, according to the previous literature [38,39,40,41,42,43,44,45,46], while the two peaks we obtained appear at 206 cm^−1^ and 617 cm^−1^, respectively, which indicates that it is possible that the ion adsorption weakened the out-of-plane motion of Ti2 and enhanced the out-of-plane motion of the C atom. According to the Raman spectra, there is no new peak in Ti_3_C_2_ doped with metal ions, and we can confirm that no new covalent bond vibration modes are generated. Moreover, combined with SEM (Figure 3), we can prove that the metal ions have been tightly bound to Ti_3_C_2_ by physical adsorption.

**Figure 6:**
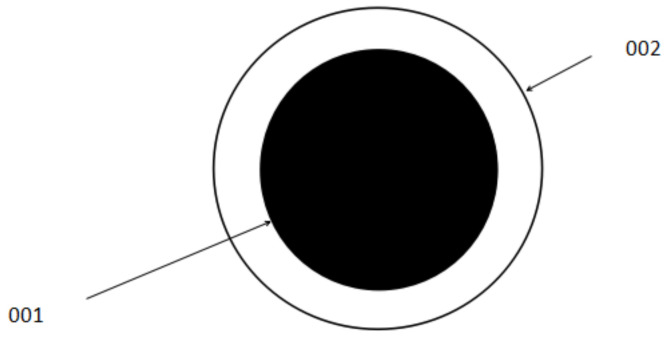
Schematic diagram of a thin film of Ti_3_C_2_ or Ti_3_C_2_:Fe^3+^/Co^2+^/Ni^2+^ powders obtained through vacuum filtration (001 is a Ti_3_C_2_/metal ion film, and 002 is a nylon filter membrane).

**Figure 7:**
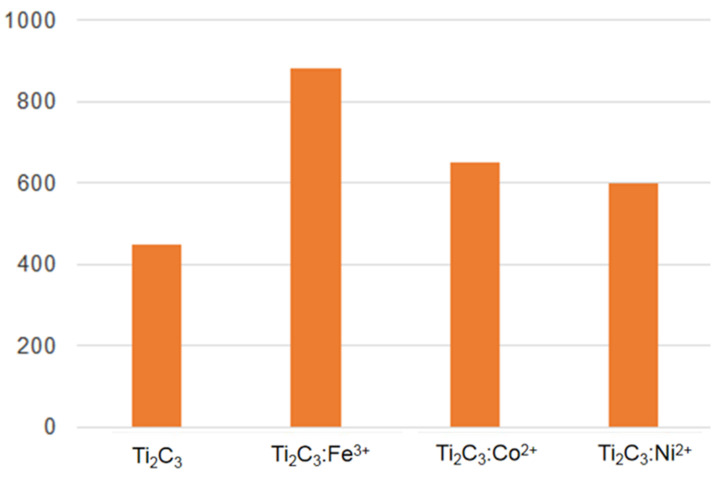
The electrical conductivity of Ti_3_C_2_, Ti_3_C_2_/Fe^3+^/Co^2+^/Ni^2+^ films.

For convenience of EMI shielding testing, Ti_3_C_2_ and Ti_3_C_2_:Fe^3+^/Co^2+^/Ni^2+^ powders are made into thin films through vacuum filtration, and a schematic diagram of a thin film is shown in Figure 6. First, the thicknesses of Ti_3_C_2_, Ti_3_C_2_/Fe^3+^/Co^2+^/Ni^2+^ films is measured, which have the same thickness, all are 0.05 mm. Then, since electrical conductivity is an important factor in EMI shielding, the electrical conductivity of Ti_3_C_2_, Ti_3_C_2_/Fe^3+^/Co^2+^/Ni^2+^ films is tested, and the corresponding values are 450, 880, 650, and 600 Ms·cm^−^^1^, as shown in Figure 7. It shows that the electrical conductivity increases when Ti_3_C_2_ is adsorbed by Fe^3+^/Co^2+^/Ni^2+^ ions, because the metal ions adsorption can enhance the conductivity of the materials. The EMI shielding performance of the materials is tested by the coaxial transmission/reflection method. The film is fixed at the connection of the two transmission lines. By transmitting the set electromagnetic wave, the S parameter data of the four materials in the frequency range of 8–18 GHz can be obtained. Using the equivalent S parameter, the transmittance (T), reflectance (R), and absorbance (A) of the tested material can be obtained. The ratio of incident electromagnetic wave power (P_O_) and transmitted electromagnetic wave power (P_I_) can be calculated, and the EMI SE of the materials can be obtained. The specific formulas are as follows [49,50,51]:

(1) $$T\;=\;10^{\frac{S_{21}}{10}}$$

(2) $$R\;=\;10^{\frac{S_{11}}{10}}$$

(3) A = 1 − R − T

(4) $$\mathrm{SE}\;=\;\left|S_{21}\right|\;=\;-10\log\left(\frac{P_{O}}{P_{I}}\right)$$

**Figure 8:**
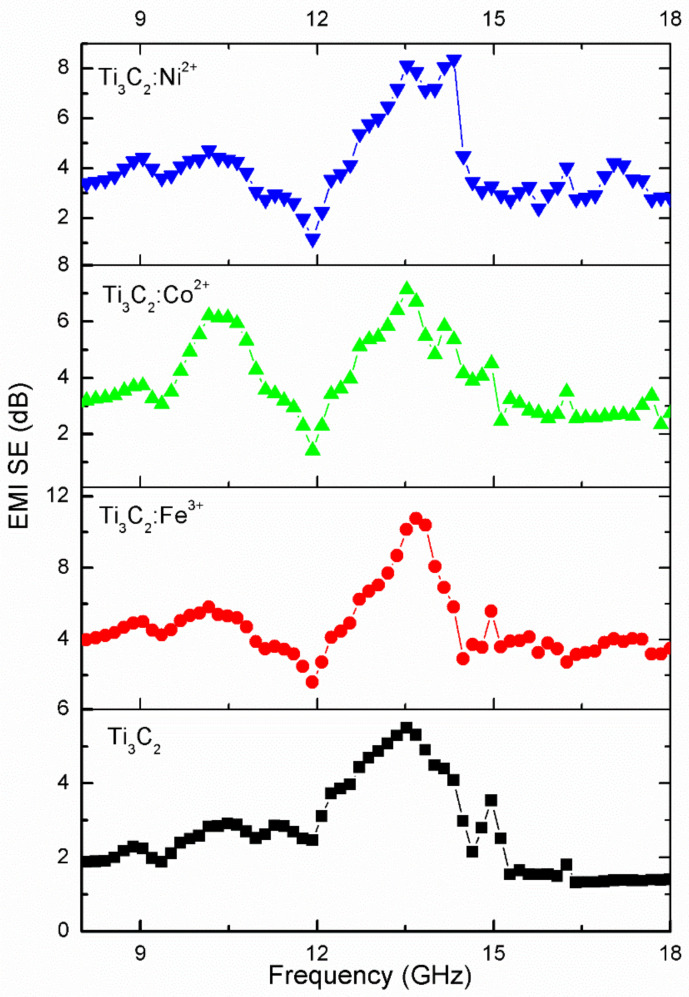
EMI SE-frequency curves of Ti_3_C_2_, Ti_3_C_2_/Fe^3+^/Co^2+^/Ni^2+^ films from 8–18 GHz.

**Figure 9:**
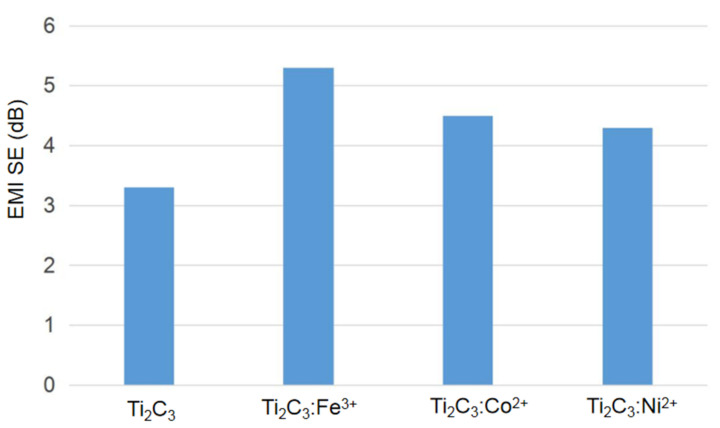
Average shielding effectiveness of Ti_3_C_2_, Ti_3_C_2_/Fe^3+^/Co^2+^/Ni^2+^ films from 8.5–15.5 GHz.

In our paper, the EMI SEs of Ti_3_C_2_ and Ti_3_C_2_/Fe^3+^/Co^2+^/Ni^2+^ films are studied in the frequency range of 8–18 GHz, and the EMI SE-frequency curves are shown in Figure 8. We can observe that the EMI SEs are improved to different degrees when Ti_3_C_2_ is adsorbed by Fe^3+^/Co^2+^/Ni^2+^ ions, which is consistent with the electrical conductivity phenomenon. The EMI shielding increased with the increase of the electrical conductivity of Ti_3_C_2_/Fe^3+^/Co^2+^/Ni^2+^ films, and the conductive network attenuated the incident electromagnetic energy through the flow of electrons and internal scattering at the numerous interfaces. Furthermore, the average EMI SEs of the four materials at 8.5–15.5 GHz can be calculated as 3.35, 5.30, 4.51, and 4.32 dB, and the histogram is shown in Figure 9. The most obvious effect is observed for the Ti_3_C_2_:Fe^3+^ film. As we know, there are many factors that affect EMI SE, such as structural forms, composition, thickness, electrical conductivity, and so on [37]. Table 1 shows the EMI shielding performance of various structural forms based on MXenes.

## 4. Conclusions

In summary, this paper proposes a method for fabricating composites by adsorbing Fe^3+^/Co^2+^/Ni^2+^ ions into layered Ti_3_C_2_. The metal ions adsorption can improve the electrical conductivity in the materials, and the conductive network attenuated the incident electromagnetic energy through the flow of electrons and internal scattering at the numerous interfaces. Therefore, the Ti_3_C_2_:Fe^3+^/Co^2+^/Ni^2+^ composites have a better EMI shielding effectiveness than the pure Ti_3_C_2_ material. These good EMI shielding materials can potentially be used in communications, electronics, military, and other applications.

## Figures and Tables

**Table 1:** EMI shielding performance of various structural forms based on MXenes.

Structural Forms	Composition	Thickness (mm)	SE (dB)	Ref.
Compact & laminates structures	Ti_3_C_2_T_x_	0.045	92	[36]
	Ti_3_C_2_T_x_/CNF	0.047	24	[27]
	Ti_3_C_2_T_x_/Ni	1.3	33.8	[52]
Layer-by-layer	Ti_3_C_2_T_x_/CNT	0.0002	2.9	[53]
	Ti_3_C_2_T_x_/PPy	0.45	42	[54]
Porous	Ti_3_C_2_T_x_	1	70.6	[55]
	Ti_3_C_2_T_x_/PVA	5	33	[56]
Segregated	Ti_3_C_2_T_x_/NR	0.25	53.6	[57]

## Data Availability

Data is contained within the article.
